# Supplementary figures and images for: Specific modulation of CRISPR transcriptional activators through RNA-sensing guide RNAs in mammalian cells and zebrafish embryos
Source: eLife. 2025 Jul 29;12:RP87722. doi: 10.7554/eLife.87722 (PMC12306969; doi:10.7554/eLife.87722)

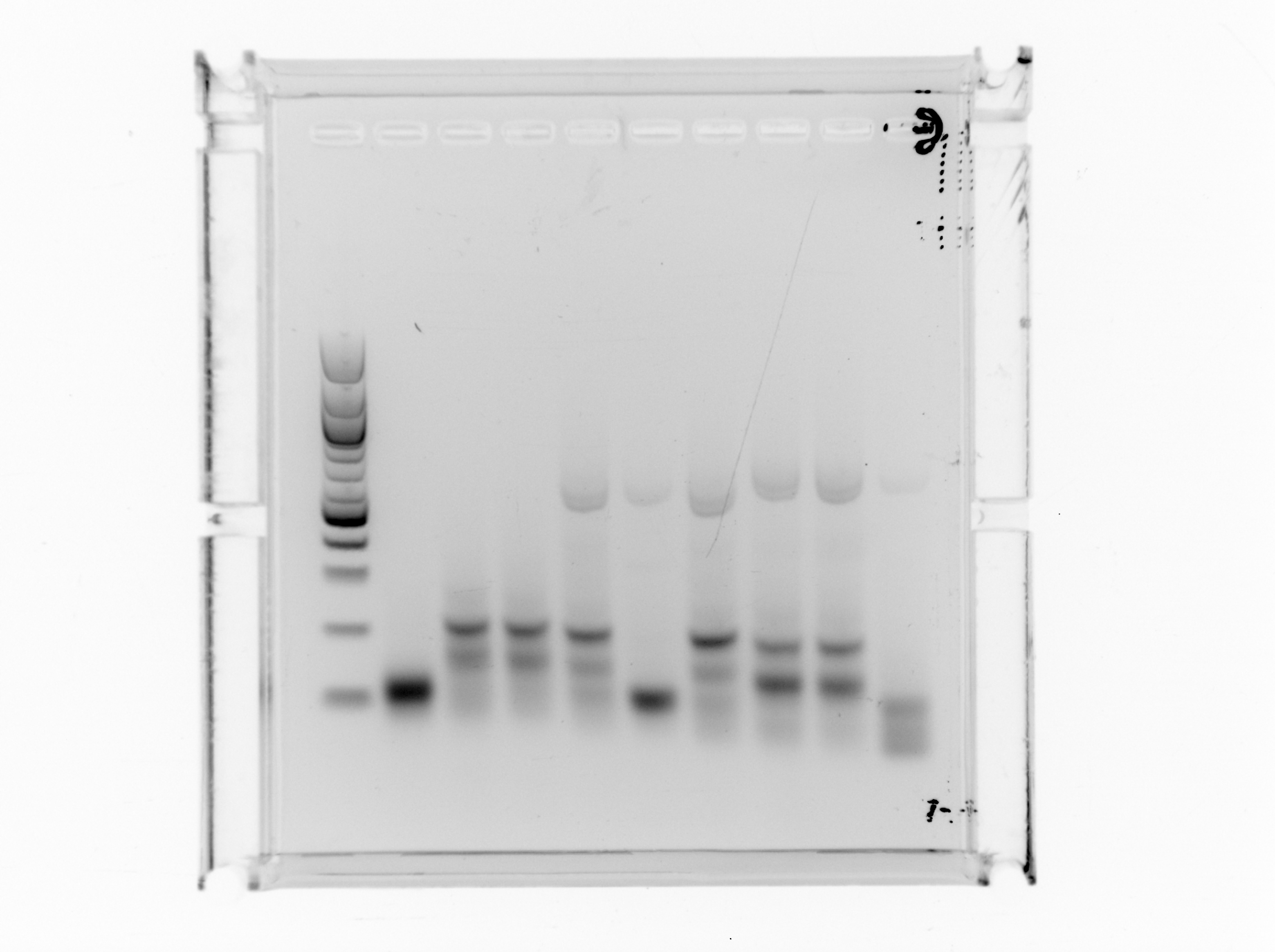

Supplement: Figure 4—source data 1. [file elife-87722-fig4-data1.zip › Figure 4- source data 1/Figure 4e- trigger circularisation assay.tif]

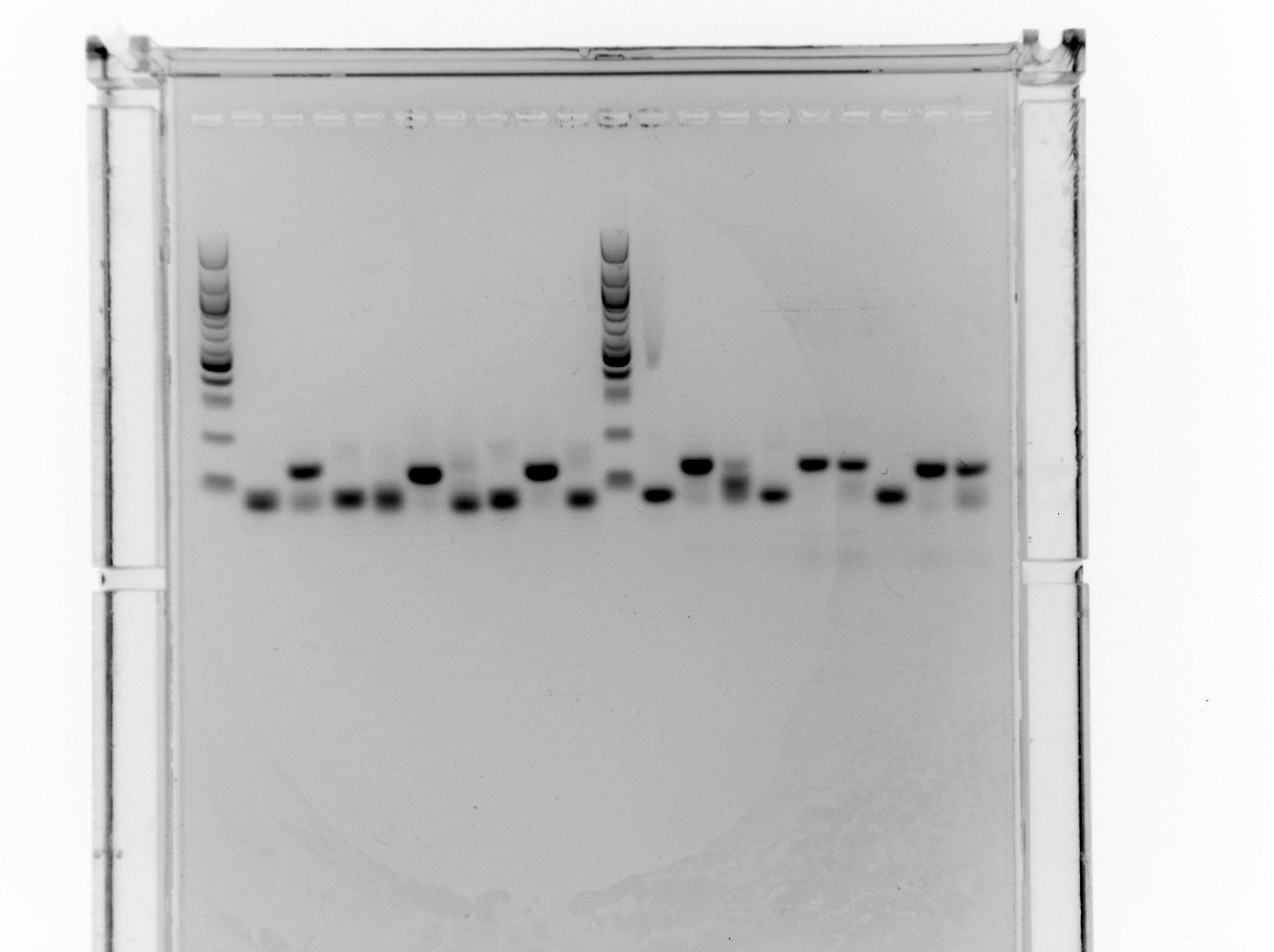

Supplement: Figure 4—source data 1. [file elife-87722-fig4-data1.zip › Figure 4- source data 1/Figure 4c- iSBH circularisation assay.tif]

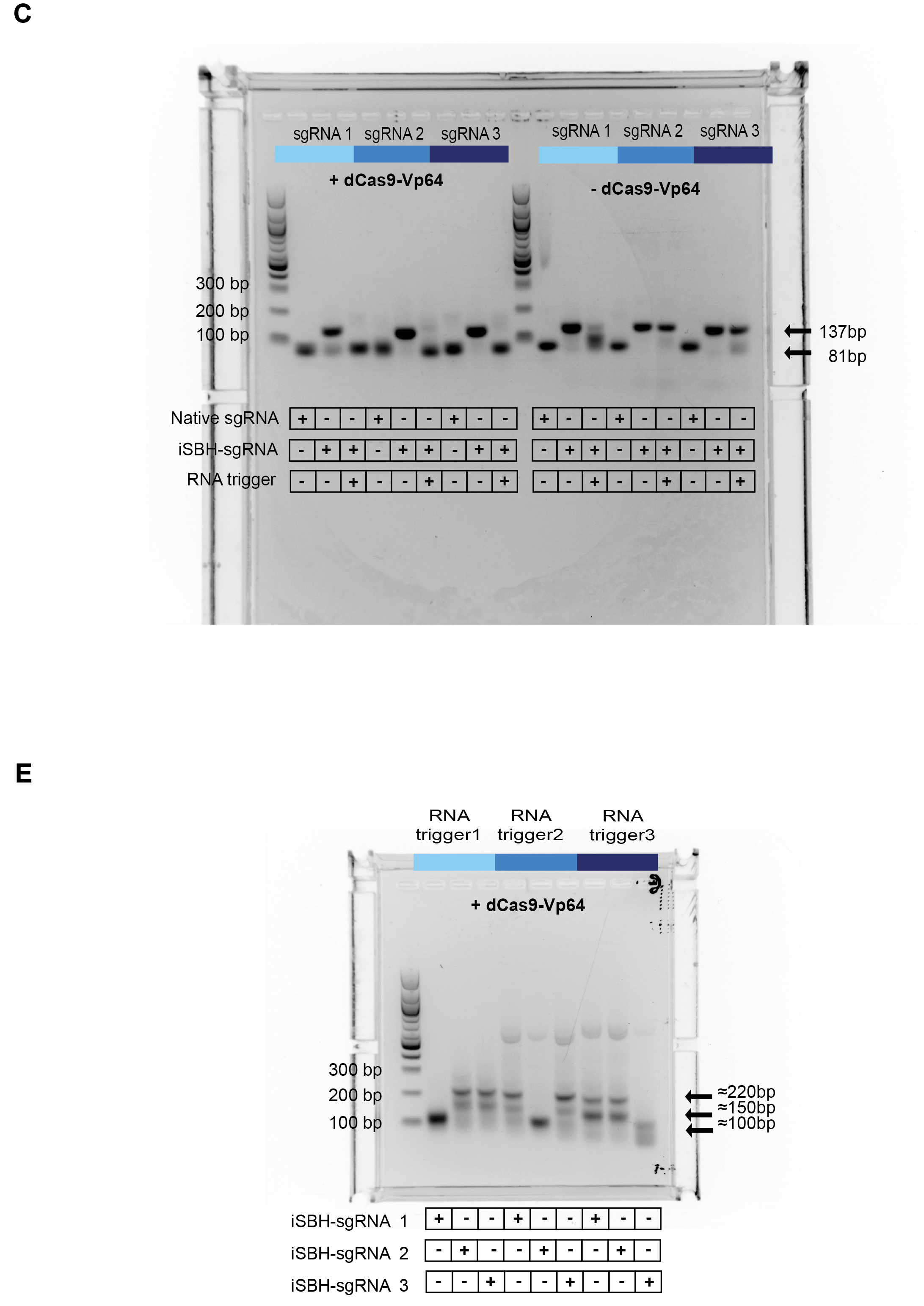

Supplement: Figure 4—source data 2. [file elife-87722-fig4-data2.zip › Figure 4- source data 2/Pelea_Figure_4_labeled_gels.png]
