# Supplementary material for: Specific modulation of CRISPR transcriptional activators through RNA-sensing guide RNAs in mammalian cells and zebrafish embryos
Source: eLife. 2025 Jul 29;12:RP87722. doi: 10.7554/eLife.87722 (PMC12306969; doi:10.7554/eLife.87722)
Supplement: Supplementary file 1. [file elife-87722-supp1.pdf]

Specific Modulation of CRISPR Transcriptional Activators through RNA-Sensing Guide RNAs in Mammalian Cells and Zebrafish Embryos

Oana Pelea[1, ‡, *], **Sarah Mayes**[1], **Quentin RV. Ferry**[1, §], **Tudor A. Fulga**[1, ¶], **Tatjana Sauka-Spengler**[1,2,*]

[1]University of Oxford, MRC Weatherall Institute of Molecular Medicine, Radcliffe Department of Medicine, Oxford OX3 9DS, United Kingdom; [2]Stowers Institute for Medical Research, Kansas City, MO 64110, United States

Supplementary Materials

File includes all relevant RNA sequences and primers

Native sgRNA sequences

+G Spacer scaffold

sgRNA 1:
GCAGTCGCGTGTAGCGAAGCAGTTTTAGAGCTAGAAATAGCAAGTTAAAATAAG
GCTAGTCCGTTATCAACTTGAAAAAGTGGCACCGAGTCGGTGC

sgRNA 2:
GTAAGTCGGAGTACTGTCCTGTTTTAGAGCTAGAAATAGCAAGTTAAAATAAGG
CTAGTCCGTTATCAACTTGAAAAAGTGGCACCGAGTCGGTGC

sgRNA 3:
GCATGACATTATTCCCGCCTCGTTTTAGAGCTAGAAATAGCAAGTTAAAATAAG
GCTAGTCCGTTATCAACTTGAAAAAGTGGCACCGAGTCGGTGC

sgRNA 4:
GCCAGGTAATGACTAGCAGGAGTTTTAGAGCTAGAAATAGCAAGTTAAAATAAG
GCTAGTCCGTTATCAACTTGAAAAAGTGGCACCGAGTCGGTGC

sgRNA 5:
GCACTACTTCAGTAAGAGTCGTTTTAGAGCTAGAAATAGCAAGTTAAAATAAGG
CTAGTCCGTTATCAACTTGAAAAAGTGGCACCGAGTCGGTGC

*For sgRNAs with spacer sequences starting with an A, C or T, an extra G was added for facilitating expression from U6 vectors

Native sgRNAs with randomised spacer sequences

+G randomised nt Spacer scaffold

sgRNA 1 (D1):
GAGTCGCGTGTAGCGAAGCAGTTTTAGAGCTAGAAATAGCAAGTTAAAATAAGG
CTAGTCCGTTATCAACTTGAAAAAGTGGCACCGAGTCGGTGC

sgRNA 1 (D2):
GTGGTCGCGTGTAGCGAAGCAGTTTTAGAGCTAGAAATAGCAAGTTAAAATAAG
GCTAGTCCGTTATCAACTTGAAAAAGTGGCACCGAGTCGGTGC

sgRNA 1 (D3):
GTGATCGCGTGTAGCGAAGCAGTTTTAGAGCTAGAAATAGCAAGTTAAAATAAG
GCTAGTCCGTTATCAACTTGAAAAAGTGGCACCGAGTCGGTGC

sgRNA 2 (D1):
GCTAAGTCGGAGTACTGTCCTGTTTTAGAGCTAGAAATAGCAAGTTAAAATAAG
GCTAGTCCGTTATCAACTTGAAAAAGTGGCACCGAGTCGGTGC

sgRNA 2 (D2):
GCAAAGTCGGAGTACTGTCCTGTTTTAGAGCTAGAAATAGCAAGTTAAAATAAG
GCTAGTCCGTTATCAACTTGAAAAAGTGGCACCGAGTCGGTGC

sgRNA 2 (D3):
GCATAGTCGGAGTACTGTCCTGTTTTAGAGCTAGAAATAGCAAGTTAAAATAAG
GCTAGTCCGTTATCAACTTGAAAAAGTGGCACCGAGTCGGTGC

sgRNA 3 (D1):
GATGACATTATTCCCGCCTCGTTTTAGAGCTAGAAATAGCAAGTTAAAATAAGG
CTAGTCCGTTATCAACTTGAAAAAGTGGCACCGAGTCGGTGC

sgRNA 3 (D2):
GGTGACATTATTCCCGCCTCGTTTTAGAGCTAGAAATAGCAAGTTAAAATAAGG
CTAGTCCGTTATCAACTTGAAAAAGTGGCACCGAGTCGGTGC

sgRNA 3 (D3):
GGGGACATTATTCCCGCCTCGTTTTAGAGCTAGAAATAGCAAGTTAAAATAAGG
CTAGTCCGTTATCAACTTGAAAAAGTGGCACCGAGTCGGTGC

First-generation iSBH-sgRNA sequences

+GC spacer* loop spacer scaffold

*An additional GC sequence was added to all iSBH-sgRNA sequences. G helps expression from U6 promoter, while C base-pairs with the first C from the scaffold

First-generation iSBH-sgRNA 1:
GCTGCTTCGCTAGTCGCTTCTGCACACTACGCATCCCAGTCGCGTGTAGCGAA
GCAGTTTTAGAGCTAGAAATAGCAAGTTAAAATAAGGCTAGTCCGTTATCAACT
TGAAAAAGTGGCACCGAGTCGGTGC

First-generation iSBH-sgRNA 2:
GCAGGACAGTACAGCGAGATACGAACATCCCTAACAGTAAGTCGGAGTACTGT
CCTGTTTTAGAGCTAGAAATAGCAAGTTAAAATAAGGCTAGTCCGTTATCAACT
TGAAAAAGTGGCACCGAGTCGGTGC

First-generation iSBH-sgRNA 3:
GCGAGGCGGGAAGTATGGAATGGACCAGTGAATACACATGACATTATTCCCGC
CTCGTTTTAGAGCTAGAAATAGCAAGTTAAAATAAGGCTAGTCCGTTATCAACT
TGAAAAAGTGGCACCGAGTCGGTGC

First-generation iSBH-sgRNA 4:
GCTCCTGCTAGTACTTAAATGGGCTCCCCACATCAACCAGGTAATGACTAGCAG
GAGTTTTAGAGCTAGAAATAGCAAGTTAAAATAAGGCTAGTCCGTTATCAACTTG
AAAAAGTGGCACCGAGTCGGTGC

First-generation iSBH-sgRNA 5:
GCGACTCTTACTCCAGTTCTGCATCTTGTTCGTCCAGCACTACTTCAGTAAGAG
TCGTTTTAGAGCTAGAAATAGCAAGTTAAAATAAGGCTAGTCCGTTATCAACTTG
AAAAAGTGGCACCGAGTCGGTGC

Second-generation iSBH-sgRNA sequences

+GC spacer* extension* loop extension spacer scaffold
Backfold=spacer*+extension+loop

*An additional GC sequence was added to all iSBH-sgRNA sequences. G helps expression from U6 promoter, while C base-pairs with the first C from the scaffold

Second-generation iSBH-sgRNA 1:
GCTGCTTCGCTAGTCGCTTCTGTCCGTACGTTCACACTACGCATCCAACTGACG
TTCAGTCGCGTGTAGCGAAGCAGTTTTAGAGCTAGAAATAGCAAGTTAAAATAA
GGCTAGTCCGTTATCAACTTGAAAAAGTGGCACCGAGTCGGTGC

Second-generation iSBH-sgRNA 2:
GCAGGACAGTACAGCGAGATACCAGCTTAGTCGAACATCCCTAACAGACATAG
CCCGTAAGTCGGAGTACTGTCCTGTTTTAGAGCTAGAAATAGCAAGTTAAAATA
AGGCTAGTCCGTTATCAACTTGAAAAAGTGGCACCGAGTCGGTGC

Second-generation iSBH-sgRNA 3:
GCGAGGCGGGAAGTATGGAATGAGTGGGATGGGACCAGTGAATACACCAAAC
CAGACATGACATTATTCCCGCCTCGTTTTAGAGCTAGAAATAGCAAGTTAAAAT
AAGGCTAGTCCGTTATCAACTTGAAAAAGTGGCACCGAGTCGGTGC

Modular iSBH-sgRNA sequences

+GC spacer* trigger-sensing region extension spacer scaffold

*An additional GC sequence was added to all iSBH-sgRNA sequences. G helps expression from U6 promoter, while C base-pairs with the first C from the scaffold

Modular iSBH-sgRNA (trigger D/sgRNA2/loop=14nt):
GCAGGACAGTACAGCGAGACAGTGTTCTCTGCACAGATAAGGACAAGCAATGA
AATCTGAGTCGGAGTACTGTCCTGTTTTAGAGCTAGAAATAGCAAGTTAAAATAA
GGCTAGTCCGTTATCAACTTGAAAAAGTGGCACCGAGTCGGTGC

Modular iSBH-sgRNA (trigger D/sgRNA3/loop=14nt):
GCGAGGCGGGAAGTATGGACAGTGTTCTCTGCACAGATAAGGACAAGCATTGA
AATCTGGACATTATTCCCGCCTCGTTTTAGAGCTAGAAATAGCAAGTTAAAATAA
GGCTAGTCCGTTATCAACTTGAAAAAGTGGCACCGAGTCGGTGC

Modular iSBH-sgRNA (trigger D/sgRNA5/loop=14nt):
GCGACTCTTACTCCAGTCAGTGTTCTCTGCACAGATAAGGACAAACGTGATGAG
TTCACCTACTTCAGTAAGAGTCGTTTTAGAGCTAGAAATAGCAAGTTAAAATAAG
GCTAGTCCGTTATCAACTTGAAAAAGTGGCACCGAGTCGGTGC

Modular iSBH-sgRNA (trigger A/sgRNA2/loop=14nt (1)):
GCAGGACAGTACAACGAAAACTGATCATCACCACAACACAACTAAAGGTCTTGA
GAAGTAGTCGGAGTACTGTCCTGTTTTAGAGCTAGAAATAGCAAGTTAAAATAA
GGCTAGTCCGTTATCAACTTGAAAAAGTGGCACCGAGTCGGTGC

Modular iSBH-sgRNA (trigger A/sgRNA2/loop=14nt (2)):
GCAGGACAGTACAACGAACGACTAAACTGATCATCACCACAACACGGATATGTT
GCGTCAGTCGGAGTACTGTCCTGTTTTAGAGCTAGAAATAGCAAGTTAAAATAA
GGCTAGTCCGTTATCAACTTGAAAAAGTGGCACCGAGTCGGTGC

Modular iSBH-sgRNA (trigger A/sgRNA2/loop=16nt):
GCAGGACAGTACAGCGAACGACTAAACTGATCATCACCACAACACAACGATATG
TTGCGTCAGTCGGAGTACTGTCCTGTTTTAGAGCTAGAAATAGCAAGTTAAAAT
AAGGCTAGTCCGTTATCAACTTGAAAAAGTGGCACCGAGTCGGTGC

Modular iSBH-sgRNA (trigger A/sgRNA2/loop=18nt):
GCAGGACAGTACAGCGAACGACTAAACTGATCATCACCACAACACAACTAGATA
TGTTGCGTCAGTCGGAGTACTGTCCTGTTTTAGAGCTAGAAATAGCAAGTTAAA
ATAAGGCTAGTCCGTTATCAACTTGAAAAAGTGGCACCGAGTCGGTGC

Modular iSBH-sgRNA (trigger A/sgRNA2/loop=20nt):
GCAGGACAGTACAACGAACGACTAAACTGATCATCACCACAACACAACTAAAGA
TATGTTGCGTCAGTCGGAGTACTGTCCTGTTTTAGAGCTAGAAATAGCAAGTTA
AAATAAGGCTAGTCCGTTATCAACTTGAAAAAGTGGCACCGAGTCGGTGC

Modular iSBH-sgRNA (trigger A/sgRNA2/loop=22nt):
GCAGGACAGTACAGCGAAACACAACTAAACTGATCATCACCACAACACAACTAA
CAGCGTAGGAGTGAGTCGGAGTACTGTCCTGTTTTAGAGCTAGAAATAGCAAGT
TAAAATAAGGCTAGTCCGTTATCAACTTGAAAAAGTGGCACCGAGTCGGTGC

Modular iSBH-sgRNA (trigger A/sgRNA2/loop=24nt):
GCAGGACAGTACAGCGAAACACAACTAAACTGATCATCACCACAACACAACTAA
ACCAGCGTAGGAGTGAGTCGGAGTACTGTCCTGTTTTAGAGCTAGAAATAGCAA
GTTAAAATAAGGCTAGTCCGTTATCAACTTGAAAAAGTGGCACCGAGTCGGTGC

Modular iSBH-sgRNA (trigger A/sgRNA2/loop=26nt):
GCAGGACAGTACAGCGAAACACAACTAAACTGATCATCACCACAACACAACTAA
ACTGCAGCGTAGGAGTGAGTCGGAGTACTGTCCTGTTTTAGAGCTAGAAATAG
CAAGTTAAAATAAGGCTAGTCCGTTATCAACTTGAAAAAGTGGCACCGAGTCGG
TGC

Modular iSBH-sgRNA (trigger A/sgRNA2/loop=28nt):
GCAGGACAGTACAGCGAAAACTGATCATCACCACAACACAACTAAACTGATCAT
CACCACGGTCTTGAGAAGTAGTCGGAGTACTGTCCTGTTTTAGAGCTAGAAATA
GCAAGTTAAAATAAGGCTAGTCCGTTATCAACTTGAAAAAGTGGCACCGAGTCG
GTGC

Modular iSBH-sgRNA (trigger A/sgRNA2/loop=30nt):
GCAGGACAGTACAGCGAAAACTGATCATCACCACAACACAACTAAACTGATCAT
CACCACAAGGTCTTGAGAAGTAGTCGGAGTACTGTCCTGTTTTAGAGCTAGAAA
TAGCAAGTTAAAATAAGGCTAGTCCGTTATCAACTTGAAAAAGTGGCACCGAGT
CGGTGC

Modular iSBH-sgRNA (trigger A/sgRNA2/loop=32nt):
GCAGGACAGTACAGCGAAAACTGATCATCACCACAACACAACTAAACTGATCAT
CACCACAACAGGTCTTGAGAAGTAGTCGGAGTACTGTCCTGTTTTAGAGCTAGA
AATAGCAAGTTAAAATAAGGCTAGTCCGTTATCAACTTGAAAAAGTGGCACCGA
GTCGGTGC

Modular iSBH-sgRNA (trigger A/sgRNA2/loop=34nt):
GCAGGACAGTACAGCGAAAACTGATCATCACCACAACACAACTAAACTGATCAT
CACCACAACACAGGTCTTGAGAAGTAGTCGGAGTACTGTCCTGTTTTAGAGCTA
GAAATAGCAAGTTAAAATAAGGCTAGTCCGTTATCAACTTGAAAAAGTGGCACC
GAGTCGGTGC

Modular iSBH-sgRNA (trigger B/sgRNA2/loop=14nt (1)):
GCAGGACAGTACGACGAGATGAAGTGAAAAACAGAGGAAGGAAGGAGTTGGTC
AAGTCAAGTCGGAGTACTGTCCTGTTTTAGAGCTAGAAATAGCAAGTTAAAATAA

GGCTAGTCCGTTATCAACTTGAAAAAGTGGCACCGAGTCGGTGC

Modular iSBH-sgRNA (trigger B/sgRNA2/loop=14nt (2)):
GCAGGACAGTACAACGAACAACGACCAAATGTCTCTAGTCATCCCCACAGGTG
GAAGTTAGTCGGAGTACTGTCCTGTTTTAGAGCTAGAAATAGCAAGTTAAAATAA
GGCTAGTCCGTTATCAACTTGAAAAAGTGGCACCGAGTCGGTGC

Modular iSBH-sgRNA (trigger B/sgRNA2/loop=16nt):
GCAGGACAGTACAGCGAGGGGCAGATGAAGTGAAAAACAGAGGAAGGACACG
GCATAGGCCAGTCGGAGTACTGTCCTGTTTTAGAGCTAGAAATAGCAAGTTAAA
ATAAGGCTAGTCCGTTATCAACTTGAAAAAGTGGCACCGAGTCGGTGC

Modular iSBH-sgRNA (trigger B/sgRNA2/loop=18nt):
GCAGGACAGTACAGCGAGGGCAGATGAAGTGAAAAACAGAGGAAGGAAGGTC
AAGTCAGATGCAGTCGGAGTACTGTCCTGTTTTAGAGCTAGAAATAGCAAGTTA
AAATAAGGCTAGTCCGTTATCAACTTGAAAAAGTGGCACCGAGTCGGTGC

Modular iSBH-sgRNA (trigger B/sgRNA2/loop=20nt):
GCAGGACAGTACAGCGAGGGGCAGATGAAGTGAAAAACAGAGGAAGGAAGGA
CACGGCATAGGCCAGTCGGAGTACTGTCCTGTTTTAGAGCTAGAAATAGCAAGT
TAAAATAAGGCTAGTCCGTTATCAACTTGAAAAAGTGGCACCGAGTCGGTGC

Modular iSBH-sgRNA (trigger B/sgRNA2/loop=22nt):
GCAGGACAGTACAGCGAGATGAAGTGAAAAACAGAGGAAGGAAGGATTGTGG
GAGTTGGTCAAGTCAAGTCGGAGTACTGTCCTGTTTTAGAGCTAGAAATAGCAA
GTTAAAATAAGGCTAGTCCGTTATCAACTTGAAAAAGTGGCACCGAGTCGGTGC

Modular iSBH-sgRNA (trigger B/sgRNA2/loop=24nt):
GCAGGACAGTACAGCGAGATGGGGGCAGATGAAGTGAAAAACAGAGGAAGGA
AGGATCAGATGCAACCAAGTCGGAGTACTGTCCTGTTTTAGAGCTAGAAATAGC
AAGTTAAAATAAGGCTAGTCCGTTATCAACTTGAAAAAGTGGCACCGAGTCGGTGC

Modular iSBH-sgRNA (trigger B/sgRNA2/loop=26nt):
GCAGGACAGTACAGCGAGGCGATCACAACGACCAAATGTCTCTAGTCATCCCC
TGCTTGTCAGTGTATTCGAGTCGGAGTACTGTCCTGTTTTAGAGCTAGAAATAG
CAAGTTAAAATAAGGCTAGTCCGTTATCAACTTGAAAAAGTGGCACCGAGTCGGTGC

Modular iSBH-sgRNA (trigger B/sgRNA2/loop=28nt):
GCAGGACAGTACAGCGAGATGAAGTGAAAAACAGAGGAAGGAAGGATTGTGG
GATTATGAGTTGGTCAAGTCAAGTCGGAGTACTGTCCTGTTTTAGAGCTAGAAA
TAGCAAGTTAAAATAAGGCTAGTCCGTTATCAACTTGAAAAAGTGGCACCGAGTCGGTG
C

Modular iSBH-sgRNA (trigger C/sgRNA2/loop=14nt (1)):
GCAGGACAGTACGACGAAGACAGACTGTGAAAGGAGAGGCGAGGGATTTATCA
GGATGTAGTCGGAGTACTGTCCTGTTTTAGAGCTAGAAATAGCAAGTTAAAATA
AGGCTAGTCCGTTATCAACTTGAAAAAGTGGCACCGAGTCGGTGC

Modular iSBH-sgRNA (trigger C/sgRNA2/loop=14nt (2)):
GCAGGACAGTACGACGAAGTGCGCAGCGCGCCTCTCTCTCTCGCTCGGCAAG
CTAAGCAAGTCGGAGTACTGTCCTGTTTTAGAGCTAGAAATAGCAAGTTAAAAT
AAGGCTAGTCCGTTATCAACTTGAAAAAGTGGCACCGAGTCGGTGC

Modular iSBH-sgRNA (trigger C/sgRNA2/loop=16nt):
GCAGGACAGTACAGCGAACAGACAGACTGTGAAAGGAGAGGCGAGGGATCAA

TGTCAATCTAGTCGGAGTACTGTCCTGTTTTAGAGCTAGAAATAGCAAGTTAAAA
TAAGGCTAGTCCGTTATCAACTTGAAAAAGTGGCACCGAGTCGGTGC

Modular iSBH-sgRNA (trigger C/sgRNA2/loop=18nt):
GCAGGACAGTACAGCGAACAGACAGACTGTGAAAGGAGAGGCGAGGGAGGTC
AATGTCAATCTAGTCGGAGTACTGTCCTGTTTTAGAGCTAGAAATAGCAAGTTAA
AATAAGGCTAGTCCGTTATCAACTTGAAAAAGTGGCACCGAGTCGGTGC

Modular iSBH-sgRNA (trigger C/sgRNA2/loop=20nt):
GCAGGACAGTACAGCGAACAGACAGACTGTGAAAGGAGAGGCGAGGGAGGAG
TCAATGTCGTTCTAGTCGGAGTACTGTCCTGTTTTAGAGCTAGAAATAGCAAGTT
AAAATAAGGCTAGTCCGTTATCAACTTGAAAAAGTGGCACCGAGTCGGTGC

Modular iSBH-sgRNA (trigger C/sgRNA2/loop=22nt):
GCAGGACAGTACAGCGAACAGACAGACTGTGAAAGGAGAGGCGAGGGAGGAG
GGTCAATGTCAATCTAGTCGGAGTACTGTCCTGTTTTAGAGCTAGAAATAGCAA
GTTAAAATAAGGCTAGTCCGTTATCAACTTGAAAAAGTGGCACCGAGTCGGTGC

Modular iSBH-sgRNA (trigger C/sgRNA2/loop=24nt):
GCAGGACAGTACAGCGAAGACAGACTGTGAAAGGAGAGGCGAGGGAGGAGGG
ATGATTTATCAGGATGTAGTCGGAGTACTGTCCTGTTTTAGAGCTAGAAATAGC
AAGTTAAAATAAGGCTAGTCCGTTATCAACTTGAAAAAGTGGCACCGAGTCGGT
GC

Modular iSBH-sgRNA (trigger C/sgRNA2/loop=26nt):
GCAGGACAGTACAGCGAACAGACAGACTGTGAAAGGAGAGGCGAGGGAGGAG
GGATGATCAATGTCGTTCTAGTCGGAGTACTGTCCTGTTTTAGAGCTAGAAATA
GCAAGTTAAAATAAGGCTAGTCCGTTATCAACTTGAAAAAGTGGCACCGAGTCG
GTGC

Modular iSBH-sgRNA (trigger C/sgRNA2/loop=32nt):
GCAGGACAGTACAGCGAGACTTTGACAGACAGACTGTGAAAGGAGAGGCGAG
GGAGGAGGGATGCTGGATGTATAAGAGTCGGAGTACTGTCCTGTTTTAGAGCT
AGAAATAGCAAGTTAAAATAAGGCTAGTCCGTTATCAACTTGAAAAAGTGGCAC
CGAGTCGGTGC

Modular iSBH-sgRNA (trigger D/sgRNA2/loop=14nt (1)):
GCAGGACAGTACAGCGAGACAGTGTTCTCTGCACAGATAAGGACAAGCAATGA
AATCTGAGTCGGAGTACTGTCCTGTTTTAGAGCTAGAAATAGCAAGTTAAAATAA
GGCTAGTCCGTTATCAACTTGAAAAAGTGGCACCGAGTCGGTGC

Modular iSBH-sgRNA (trigger D/sgRNA2/loop=14nt (2)):
GCAGGACAGTACAGCGAAGTGTTCTCTGCACAGATAAGGACAAACATGTTAAGA
CTACAAGTCGGAGTACTGTCCTGTTTTAGAGCTAGAAATAGCAAGTTAAAATAA
GGCTAGTCCGTTATCAACTTGAAAAAGTGGCACCGAGTCGGTGC

Modular iSBH-sgRNA (trigger D/sgRNA2/loop=16nt):
GCAGGACAGTACAGCGAGACAGTGTTCTCTGCACAGATAAGGACAAACGCAAT
GAAATCTGAGTCGGAGTACTGTCCTGTTTTAGAGCTAGAAATAGCAAGTTAAAA
TAAGGCTAGTCCGTTATCAACTTGAAAAAGTGGCACCGAGTCGGTGC

Modular iSBH-sgRNA (trigger D/sgRNA2/loop=18nt):
GCAGGACAGTACAGCGAAGACAGTGTTCTCTGCACAGATAAGGACAAACACAG
TTAACTATGTAGTCGGAGTACTGTCCTGTTTTAGAGCTAGAAATAGCAAGTTAAA
ATAAGGCTAGTCCGTTATCAACTTGAAAAAGTGGCACCGAGTCGGTGC

Modular iSBH-sgRNA (trigger D/sgRNA2/loop=20nt):
GCAGGACAGTACAGCGAAGCAAATGTGTTGCCAAAAAGGATGCTTTAGAGACG
GCTTCACTGTTGAGTCGGAGTACTGTCCTGTTTTAGAGCTAGAAATAGCAAGTT
AAAATAAGGCTAGTCCGTTATCAACTTGAAAAAGTGGCACCGAGTCGGTGC

Modular iSBH-sgRNA (trigger D/sgRNA2/loop=22nt):
GCAGGACAGTACAGCGAGAGACAGTGTTCTCTGCACAGATAAGGACAAACATT
AAGATTACAAGGTCAGTCGGAGTACTGTCCTGTTTTAGAGCTAGAAATAGCAAG
TTAAAATAAGGCTAGTCCGTTATCAACTTGAAAAAGTGGCACCGAGTCGGTGC

Modular iSBH-sgRNA (trigger D/sgRNA2/loop=24nt):
GCAGGACAGTACAGCGAAGAGACAGTGTTCTCTGCACAGATAAGGACAAACAT
TATGAGTTCACGATCTAGTCGGAGTACTGTCCTGTTTTAGAGCTAGAAATAGCA
AGTTAAAATAAGGCTAGTCCGTTATCAACTTGAAAAAGTGGCACCGAGTCGGTG
C

Modular iSBH-sgRNA (trigger D/sgRNA2/loop=34nt):
GCAGGACAGTACAGCGAGACAGTGTTCTCTGCACAGATAAGGACAAACATTATT
CAGAGGGAGTACGCATTGAAATCTGAGTCGGAGTACTGTCCTGTTTTAGAGCTA
GAAATAGCAAGTTAAAATAAGGCTAGTCCGTTATCAACTTGAAAAAGTGGCACC
GAGTCGGTGC

*An additional GC sequence was added to all iSBH-sgRNA sequences. G helps expression from U6 promoter, while C base-pairs with the first C from the scaffold

RNA triggers for first-generation iSBH-sgRNAs

hairpin linker 34nt trigger 100nt 3' flank linker hairpin
random sequence

Non-complementary RNA trigger (transfected in all OFF states):
GGCTCGAAAGAGCCACGGGTCTTCGAGCATTGGAAGTCTAGATACCTGCTTTA
CGAAGACCTATGCTAGTACGAAAGTACTAGC

First-generation iSBH-sgRNA 1 trigger:
GGCTCGAAAGAGCCACGGATGCGTAGTGTGCAGAAGCGACTAGCGAAGCAAT
GCTAGTACGAAAGTACTAGC

First-generation iSBH-sgRNA 2 trigger:
GGCTCGAAAGAGCCACTGTTAGGGATGTTCGTATCTCGCTGTACTGTCCTGCAT
GCTAGTACGAAAGTACTAGC
*GC complementary with extra GC sequences on the iSBH-sgRNA 1

First-generation iSBH-sgRNA 3 trigger:
GGCTCGAAAGAGCCACTGTATTCACTGGTCCATTCCATACTTCCCGCCTCATGC
TAGTACGAAAGTACTAGC

First-generation iSBH-sgRNA 4 trigger:
GGCTCGAAAGAGCCACTTGATGTGGGGAGCCCATTTAAGTACTAGCAGGAATG
CTAGTACGAAAGTACTAGC

First-generation iSBH-sgRNA 5 trigger:

GGCTCGAAAGAGCCACTGGACGAACAAGATGCAGAACTGGAGTAAGAGTCATG
CTAGTACGAAAGTACTAGC

First-generation iSBH-sgRNA 1 trigger 100nt 3' flank:
GGCTCGAAAGAGCCACTGAGACGTAAGACTCGAGCGGTCCGTCTCAGTCAGTA
TGGTCTTGGGATGCGTAGTGTGCAGAAGCGACTAGCGAAGCAGGCGGCCGTA
TGAGGGACAATTGGAGaagtgaattatataaatataaagtagtaaaaattgaaccattaggagtagcacc
caccaagGCAAAGAGAAGAGTGGTGCAGGTACGAAAGTAC

First-generation iSBH-sgRNA 2 trigger 100nt 3' flank:
GGCTCGAAAGAGCCACTGAGACGTAAGACTCGAGCGGTCCGTCTCAGTCATAG
TATATGTGTGTTAGGGATGTTCGTATCTCGCTGTACTGTCCTGGCGGCCGCATA
GTTAAGCCAGTATCTGCtccctgcttgtgtgttggaggtcgctgagtagtgcgcgagcaaaatttaagctac
aacaaGGCAAGGCTTGACCGACAAGTACGAAAGTAC

First-generation iSBH-sgRNA 3 trigger 100nt 3' flank:
GGCTCGAAAGAGCCACTGAGACGTAAGACTCGAGCGGTCCGTCTCAGTCACTA
TCTATATCTGTATTCACTGGTCCATTCCATACTTCCCGCCTCGGCGGCCGTCGG
GAGATCTCCCGATCCCCtatggtgcactctcagtacaatctgctctgatgccgcatagttaagccagtatct
GCTCCCTGCTTGTGTGTTGGAGGTGTACGAAAGTAC

RNA triggers for second-generation iSBH-sgRNAs

hairpin linker 100nt 5' flank 44nt trigger 100nt 3' flank linker hairpin

Second-generation iSBH-sgRNA 1 trigger:
GGCTCGAAAGAGCCACGGATGCGTAGTGTGAACGTACGGACAGAAGCGACTA
GCGAAGCAATGCTAGTACGAAAGTACTAGC

Second-generation iSBH-sgRNA 2 trigger:
GGCTCGAAAGAGCCACTGTTAGGGATGTTCGACTAAGCTGGTATCTCGCTGTA
CTGTCCTATGCTAGTACGAAAGTACTAGC

Second-generation iSBH-sgRNA 3 trigger:
GGCTCGAAAGAGCCACTGTATTCACTGGTCCCATCCCACTCATTCCATACTTCC
CGCCTCATGCTAGTACGAAAGTACTAGC

Second-generation iSBH-sgRNA 1 trigger 100nt 3' flank:
GGCTCGAAAGAGCCACTGAGACGTAAGACTCGAGCGGTCCGTCTCAGTCAGTA
TGGTCTTGGGATGCGTAGTGTGAACGTACGGACAGAAGCGACTAGCGAAGCAG
CGGCCGTATGAGGGACAATTGGAGaagtgaattatataaatataaagtagtaaaaattgaaccatta
ggagtagcacccaccaagGCAAAGAGAAGAGTGGTGCAGGTACGAAAGTAC

Second-generation iSBH-sgRNA 2 trigger 100nt 3' flank:
GGCTCGAAAGAGCCACTGAGACGTAAGACTCGAGCGGTCCGTCTCAGTCATAG
TATATGTGTGTTAGGGATGTTCGACTAAGCTGGTATCTCGCTGTACTGTCCTGC
GGCCGCATAGTTAAGCCAGTATCTGCtccctgcttgtgtgttggaggtcgctgagtagtgcgcgagc
aaaatttaagctacaacaaGGCAAGGCTTGACCGACAAGTACGAAAGTAC

Second-generation iSBH-sgRNA 3 trigger 100nt 3' flank:
GGCTCGAAAGAGCCACTGAGACGTAAGACTCGAGCGGTCCGTCTCAGTCATCG

GTCTTGTCTGTATTCACTGGTCCCATCCCACTCATTCCATACTTCCCGCCTCGC
GGCCGTCGGGAGATCTCCCGATCCCCtatggtgcactctcagtacaatctgctctgatgccgcatag
ttaagccagtatctGCTCCCTGCTTGTGTGTTGGAGGTGTACGAAAGTAC

Second-generation iSBH-sgRNA 1 trigger 100nt 5' flank:
GGCTCGAAAGAGCCACCCTATGGTGCACTCTCAGTACaatctgctctgatgccgcatagtta
agccagtatctgctccctgcttgtgtgttggaGGTCGCTGAGTAGTGCGCGAGCGTCAGTATGG
TCTTGGGATGCGTAGTGTGAACGTACGGACAGAAGCGACTAGCGAAGCAGCG
GCCGCATGCTAGTACGAAAGTACTAGC

Second-generation iSBH-sgRNA 2 trigger 100nt 5' flank:
GGCTCGAAAGAGCCACGCAAAATTTAAGCTACAACAAGGCaaggcttgaccgacaattg
catgaagaatctgcttagggttaggcgttttgcgCTGCTTCGCGATGTACGGGCCAGGTCATAGT
ATATGTGTGTTAGGGATGTTCGACTAAGCTGGTATCTCGCTGTACTGTCCTGCG
GCCGCATGCTAGTACGAAAGTACTAGC

Second-generation iSBH-sgRNA 3 trigger 100nt 5' flank:
GGCTCGAAAGAGCCACGTCGACGGATCGGGAGATCTCCCGATCCCctatggtgcact
ctcagtacaatctgctctgatgccgcatagttaagcCAGTATCTGCTCCCTGCTTGTGTGTCATCG
GTCTTGTCTGTATTCACTGGTCCCATCCCACTCATTCCATACTTCCCGCCTCGC
GGCCGCATGCTAGTACGAAAGTACTAGC

Second-generation iSBH-sgRNA 1 trigger 100nt 5'+3' flank:
GGCTCGAAAGAGCCACCCTATGGTGCACTCTCAGTACAATCTGCTCTGATGCC
GCATAGTTAAGCCAGTATCTGCTCCCTGCTTGTGTGTTGGAGGTCGCTGAGTAG
TGCGCGAGCGTCAGTATGGTCTTGGGATGCGTAGTGTGAACGTACGGACAGAA
GCGACTAGCGAAGCAGCGGCCGTATGAGGGACAATTGGAGAAGTGAATTATAT
AAATATAAAGTAGTAAAAATTGAACCATTAGGAGTAGCACCCACCAAGGCAAAG
AGAAGAGTGGTGCAGGTACGAAAGTAC

Second-generation iSBH-sgRNA 2 trigger 100nt 5'+3' flank:
GGCTCGAAAGAGCCACGCAAAATTTAAGCTACAACAAGGCAAGGCTTGACCGA
CAATTGCATGAAGAATCTGCTTAGGGTTAGGCGTTTTGCGCTGCTTCGCGATGT
ACGGGCCAGGTCATAGTATATGTGTGTTAGGGATGTTCGACTAAGCTGGTATCT
CGCTGTACTGTCCTGCGGCCGCATAGTTAAGCCAGTATCTGCTCCCTGCTTGT
GTGTTGGAGGTCGCTGAGTAGTGCGCGAGCAAAATTTAAGCTACAACAAGGCA
AGGCTTGACCGACAAGTACGAAAGTAC

Second-generation iSBH-sgRNA 3 trigger 100nt 5'+3' flank:
GGCTCGAAAGAGCCACGTCGACGGATCGGGAGATCTCCCGATCCCCTATGGT
GCACTCTCAGTACAATCTGCTCTGATGCCGCATAGTTAAGCCAGTATCTGCTCC
CTGCTTGTGTGTCATCGGTCTTGTCTGTATTCACTGGTCCCATCCCACTCATTC
CATACTTCCCGCCTCGCGGCCGTCGGGAGATCTCCCGATCCCCTATGGTGCAC
TCTCAGTACAATCTGCTCTGATGCCGCATAGTTAAGCCAGTATCTGCTCCCTGC
TTGTGTGTTGGAGGTGTACGAAAGTAC

Longer RNA triggers with engineered secondary structures for second-generation iSBH-sgRNAs

hairpin linker that base-pairs with 44nt trigger 44nt trigger 100nt 3' flank linker hairpin

Second-generation iSBH-sgRNA 1 trigger 100nt 3' flank (60%):

GGCTCGAAAGAGCCACTGAGACGTAAGACTCGAGCGGTCCGTCTCAGTCAGTA
TGGTCTTGGGATGCGTAGTGTGAACGTACGGACAGAAGCGACTAGCGAAGCAG
CGGCCGTATGAGGGACAATTGGAGaagtgaattatataaatataaagtagtaaaaattgaaccatta
ggagtagcacccaccaagGCAAAGAGAAGAGTGGTGCAGGTACGAAAGTAC

Second-generation iSBH-sgRNA 2 trigger 100nt 3’ flank (60%):
GGCTCGAAAGAGCCACTGATCCCTAAGACTCGAGCGGTCCGTCTCAGTCATAG
TATATGTGTGTTAGGGATGTTCGACTAAGCTGGTATCTCGCTGTACTGTCCTGC
GGCCGCATAGTTAAGCCAGTATCTGCTCCCTGCTTGTGTGTTGGAGGTCGCTG
AGTAGTGCGCGAGCAAAATTTAAGCTACAACAAGGCAAGGCTTGACCGACAAG
TACGAAAGTAC

Second-generation iSBH-sgRNA 3 trigger 100nt 3’ flank (60%):
GGCTCGAAAGAGCCACTGAGTGAATAGACTCGAGCGGTCCGTCTCAGTCATCG
GTCTTGTCTGTATTCACTGGTCCCATCCCACTCATTCCATACTTCCCGCCTCGC
GGCCGTCGGGAGATCTCCCGATCCCCTATGGTGCACTCTCAGTACAATCTGCT
CTGATGCCGCATAGTTAAGCCAGTATCTGCTCCCTGCTTGTGTGTTGGAGGTGT
ACGAAAGTAC

Second-generation iSBH-sgRNA 1 trigger 100nt 3’ flank (80%):
GGCTCGAAAGAGCCACTGAGACACTACGCTCGAGCGGTCCGTCTCAGTCAGTA
TGGTCTTGGGATGCGTAGTGTGAACGTACGGACAGAAGCGACTAGCGAAGCAG
CGGCCGTATGAGGGACAATTGGAGAAGTGAATTATATAAATATAAAGTAGTAAA
AATTGAACCATTAGGAGTAGCACCCACCAAGGCAAAGAGAAGAGTGGTGCAGG
TACGAAAGTAC

Second-generation iSBH-sgRNA 2 trigger 100nt 3’ flank (80%):
GGCTCGAAAGAGCCACTGAGAACATCCCCTCGAGCGGTCCGTCTCAGTCATAG
TATATGTGTGTTAGGGATGTTCGACTAAGCTGGTATCTCGCTGTACTGTCCTGC
GGCCGCATAGTTAAGCCAGTATCTGCTCCCTGCTTGTGTGTTGGAGGTCGCTG
AGTAGTGCGCGAGCAAAATTTAAGCTACAACAAGGCAAGGCTTGACCGACAAG
TACGAAAGTAC

Second-generation iSBH-sgRNA 3 trigger 100nt 3’ flank (80%):
GGCTCGAAAGAGCCACTGAGACCAGTGACTCGAGCGGTCCGTCTCAGTCATCG
GTCTTGTCTGTATTCACTGGTCCCATCCCACTCATTCCATACTTCCCGCCTCGC
GGCCGTCGGGAGATCTCCCGATCCCCTATGGTGCACTCTCAGTACAATCTGCT
CTGATGCCGCATAGTTAAGCCAGTATCTGCTCCCTGCTTGTGTGTTGGAGGTGT
ACGAAAGTAC

Second-generation iSBH-sgRNA 1 trigger 100nt 3’ flank (100%):
GGCTCGAAAGAGCCACCACACTACGCATCCCGAGCGGTCCGTCTCAGTCAGTA
TGGTCTTGGGATGCGTAGTGTGAACGTACGGACAGAAGCGACTAGCGAAGCAG
CGGCCGTATGAGGGACAATTGGAGAAGTGAATTATATAAATATAAAGTAGTAAA
AATTGAACCATTAGGAGTAGCACCCACCAAGGCAAAGAGAAGAGTGGTGCAGG
TACGAAAGTAC

Second-generation iSBH-sgRNA 2 trigger 100nt 3’ flank (100%):
GGCTCGAAAGAGCCACGAACATCCCTAACACGAGCGGTCCGTCTCAGTCATAG
TATATGTGTGTTAGGGATGTTCGACTAAGCTGGTATCTCGCTGTACTGTCCTGC
GGCCGCATAGTTAAGCCAGTATCTGCTCCCTGCTTGTGTGTTGGAGGTCGCTG
AGTAGTGCGCGAGCAAAATTTAAGCTACAACAAGGCAAGGCTTGACCGACAAG
TACGAAAGTAC

Second-generation iSBH-sgRNA 3 trigger 100nt 3’ flank (100%):

GGCTCGAAAGAGCCACGACCAGTGAATACACGAGCGGTCCGTCTCAGTCATCG
GTCTTGTCTGTATTCACTGGTCCCATCCCACTCATTCCATACTTCCCGCCTCGC
GGCCGTCGGGAGATCTCCCGATCCCCTATGGTGCACTCTCAGTACAATCTGCT
CTGATGCCGCATAGTTAAGCCAGTATCTGCTCCCTGCTTGTGTGTTGGAGGTGT
ACGAAAGTAC

RNA triggers for second-generation iSBH-sgRNA expressed from CMV promoters

CMV enhancer CMV promoter linker hairpin linker trigger linker hairpin linker bGH poly(A) signal MALAT1 terminator

Second-generation iSBH-sgRNA 1 (CMV+pA) trigger:

GACATTGATTATTGACTAGTTATTAATAGTAATCAATTACGGGGTCATTAGTTCAT
AGCCCATATATGGAGTTCCGCGTTACATAACTTACGGTAAATGGCCCGCCTGGC
TGACCGCCCAACGACCCCCGCCCATTGACGTCAATAATGACGTATGTTCCCATA
GTAACGCCAATAGGGACTTTCCATTGACGTCAATGGGTGGAGTATTTACGGTAA
ACTGCCCACTTGGCAGTACATCAAGTGTATCATATGCCAAGTACGCCCCCTATT
GACGTCAATGACGGTAAATGGCCCGCCTGGCATTATGCCCAGTACATGACCTT
ATGGGACTTTCCTACTTGGCAGTACATCTACGTATTAGTCATCGCTATTACCATG
GTGATGCGGTTTTGGCAGTACATCAATGGGCGTGGATAGCGGTTTGACTCACG
GGGATTTCCAAGTCTCCACCCCATTGACGTCAATGGGAGTTTGTTTTGGCACCA
AAATCAACGGGACTTTCCAAAATGTCGTAACAACTCCGCCCCATTGACGCAAAT
GGGCGGTAGGCGTGTACGGTGGGAGGTCTATATAAGCAGAGCTCTCTGGCTAA
CTAGAGAACCCACTGCTTACTGGCTTATCGAAATTAATACGACTCACTATAGGG
AGACCCAAGCTGGCTAGCGTTTAAACTTAAGGCTCGAAAGAGCCACGGATGCG
TAGTGTGAACGTACGGACAGAAGCGACTAGCGAAGCAATGCTAGTACGAAAGT
ACTAGCTTTTTTGCTAGCAGGCATGCTGGGGGCCGCTCGAGTCTAGAGGGCCC
GTTTAAACCCGCTGATCAGCCTCGACTGTGCCTTCTAGTTGCCAGCCATCTGTT
GTTTGCCCCTCCCCCGTGCCTTCCTTGACCCTGGAAGGTGCCACTCCCACTGT
CCTTTCCTAATAAAATGAGGAAATTGCATCGCATTGTCTGAGTAGGTGTCATTCT
ATTCTGGGGGGTGGGGTGGGGCAGGACAGCAAGGGGGAGGATTGGGAAGAC
AATAGCAGGCATGCTGGGGATGCGGTGGGCTCTATGG

Second-generation iSBH-sgRNA 1 (CMV+MALAT1) trigger:

GACATTGATTATTGACTAGTTATTAATAGTAATCAATTACGGGGTCATTAGTTCAT
AGCCCATATATGGAGTTCCGCGTTACATAACTTACGGTAAATGGCCCGCCTGGC
TGACCGCCCAACGACCCCCGCCCATTGACGTCAATAATGACGTATGTTCCCATA
GTAACGCCAATAGGGACTTTCCATTGACGTCAATGGGTGGAGTATTTACGGTAA
ACTGCCCACTTGGCAGTACATCAAGTGTATCATATGCCAAGTACGCCCCCTATT
GACGTCAATGACGGTAAATGGCCCGCCTGGCATTATGCCCAGTACATGACCTT
ATGGGACTTTCCTACTTGGCAGTACATCTACGTATTAGTCATCGCTATTACCATG
GTGATGCGGTTTTGGCAGTACATCAATGGGCGTGGATAGCGGTTTGACTCACG
GGGATTTCCAAGTCTCCACCCCATTGACGTCAATGGGAGTTTGTTTTGGCACCA
AAATCAACGGGACTTTCCAAAATGTCGTAACAACTCCGCCCCATTGACGCAAAT
GGGCGGTAGGCGTGTACGGTGGGAGGTCTATATAAGCAGAGCTCTCTGGCTAA
CTAGAGAACCCACTGCTTACTGGCTTATCGAAATTAATACGACTCACTATAGGG
AGACCCAAGCTGGCTAGCGTTTAAACTTAAGGCTCGAAAGAGCCACGGATGCG
TAGTGTGAACGTACGGACAGAAGCGACTAGCGAAGCAATGCTAGTACGAAAGT
ACTAGCTTTTTTGCTAGCAGGCATGCTGGGGGCCGCGATTCGTCAGTAGGGTT
GTAAAGGTTTTTCTTTTCCTGAGAAAACAACCTTTTGTTTTCTCAGGTTTTGCTTT
TTGGCCTTTCCCTAGCTTTAAAAAAAAAAAAGCAAAAGACGCTGGTGGCTGGCA

CTCCTGGTTTCCAGGACGGGGTTCAAGTCCCTGCGGTGTCTTTGCTT

Second-generation iSBH-sgRNA 2 (CMV+pA) trigger:
GACATTGATTATTGACTAGTTATTAATAGTAATCAATTACGGGGTCATTAGTTCAT
AGCCCATATATGGAGTTCCGCGTTACATAACTTACGGTAAATGGCCCGCCTGGC
TGACCGCCCAACGACCCCCGCCCATTGACGTCAATAATGACGTATGTTCCCATA
GTAACGCCAATAGGGACTTTCCATTGACGTCAATGGGTGGAGTATTTACGGTAA
ACTGCCCACTTGGCAGTACATCAAGTGTATCATATGCCAAGTACGCCCCCTATT
GACGTCAATGACGGTAAATGGCCCGCCTGGCATTATGCCCAGTACATGACCTT
ATGGGACTTTCCTACTTGGCAGTACATCTACGTATTAGTCATCGCTATTACCATG
GTGATGCGGTTTTGGCAGTACATCAATGGGCGTGGATAGCGGTTTGACTCACG
GGGATTTCCAAGTCTCCACCCCATTGACGTCAATGGGAGTTTGTTTTGGCACCA
AAATCAACGGGACTTTCCAAAATGTCGTAACAACTCCGCCCCATTGACGCAAAT
GGGCGGTAGGCGTGTACGGTGGGAGGTCTATATAAGCAGAGCTCTCTGGCTAA
CTAGAGAACCCACTGCTTACTGGCTTATCGAAATTAATACGACTCACTATAGGG
AGACCCAAGCTGGCTAGCGTTTAAACTTAAGGCTCGAAAGAGCCACTGTTAGG
GATGTTCGACTAAGCTGGTATCTCGCTGTACTGTCCTATGCTAGTACGAAAGTA
CTAGCTTTTTTGCTAGCAGGCATGCTGGGGGCCGCTCGAGTCTAGAGGGCCCG
TTTAAACCCGCTGATCAGCCTCGACTGTGCCTTCTAGTTGCCAGCCATCTGTTG
TTTGCCCCTCCCCCGTGCCTTCCTTGACCCTGGAAGGTGCCACTCCCACTGTC
CTTTCCTAATAAAATGAGGAAATTGCATCGCATTGTCTGAGTAGGTGTCATTCTA
TTCTGGGGGGTGGGGTGGGGCAGGACAGCAAGGGGGAGGATTGGGAAGACA
ATAGCAGGCATGCTGGGGATGCGGTGGGCTCTATGG

Second-generation iSBH-sgRNA 2 (CMV+MALAT1) trigger:
GACATTGATTATTGACTAGTTATTAATAGTAATCAATTACGGGGTCATTAGTTCAT
AGCCCATATATGGAGTTCCGCGTTACATAACTTACGGTAAATGGCCCGCCTGGC
TGACCGCCCAACGACCCCCGCCCATTGACGTCAATAATGACGTATGTTCCCATA
GTAACGCCAATAGGGACTTTCCATTGACGTCAATGGGTGGAGTATTTACGGTAA
ACTGCCCACTTGGCAGTACATCAAGTGTATCATATGCCAAGTACGCCCCCTATT
GACGTCAATGACGGTAAATGGCCCGCCTGGCATTATGCCCAGTACATGACCTT
ATGGGACTTTCCTACTTGGCAGTACATCTACGTATTAGTCATCGCTATTACCATG
GTGATGCGGTTTTGGCAGTACATCAATGGGCGTGGATAGCGGTTTGACTCACG
GGGATTTCCAAGTCTCCACCCCATTGACGTCAATGGGAGTTTGTTTTGGCACCA
AAATCAACGGGACTTTCCAAAATGTCGTAACAACTCCGCCCCATTGACGCAAAT
GGGCGGTAGGCGTGTACGGTGGGAGGTCTATATAAGCAGAGCTCTCTGGCTAA
CTAGAGAACCCACTGCTTACTGGCTTATCGAAATTAATACGACTCACTATAGGG
AGACCCAAGCTGGCTAGCGTTTAAACTTAAGGCTCGAAAGAGCCACTGTTAGG
GATGTTCGACTAAGCTGGTATCTCGCTGTACTGTCCTATGCTAGTACGAAAGTA
CTAGCTTTTTTGCTAGCAGGCATGCTGGGGGCCGCGATTCGTCAGTAGGGTTG
TAAAGGTTTTTCTTTTCCTGAGAAAACAACCTTTTGTTTTCTCAGGTTTTGCTTTT
TGGCCTTTCCCTAGCTTTAAAAAAAAAAAAAGCAAAAGACGCTGGTGGCTGGCAC
TCCTGGTTTCCAGGACGGGGTTCAAGTCCCTGCGGTGTCTTTGCTT

Second-generation iSBH-sgRNA 3 (CMV+pA) trigger:
GACATTGATTATTGACTAGTTATTAATAGTAATCAATTACGGGGTCATTAGTTCAT
AGCCCATATATGGAGTTCCGCGTTACATAACTTACGGTAAATGGCCCGCCTGGC
TGACCGCCCAACGACCCCCGCCCATTGACGTCAATAATGACGTATGTTCCCATA
GTAACGCCAATAGGGACTTTCCATTGACGTCAATGGGTGGAGTATTTACGGTAA
ACTGCCCACTTGGCAGTACATCAAGTGTATCATATGCCAAGTACGCCCCCTATT
GACGTCAATGACGGTAAATGGCCCGCCTGGCATTATGCCCAGTACATGACCTT
ATGGGACTTTCCTACTTGGCAGTACATCTACGTATTAGTCATCGCTATTACCATG
GTGATGCGGTTTTGGCAGTACATCAATGGGCGTGGATAGCGGTTTGACTCACG
GGGATTTCCAAGTCTCCACCCCATTGACGTCAATGGGAGTTTGTTTTGGCACCA
AAATCAACGGGACTTTCCAAAATGTCGTAACAACTCCGCCCCATTGACGCAAAT

GGGCGGTAGGCGTGTACGGTGGGAGGTCTATATAAGCAGAGCTCTCTGGCTAA
CTAGAGAACCCACTGCTTACTGGCTTATCGAAATTAATACGACTCACTATAGGG
AGACCCAAGCTGGCTAGCGTTTAAACTTAAGGCTCGAAAGAGCCACTGTATTCA
CTGGTCCCATCCCACTCATTCCATACTTCCCGCCTCATGCTAGTACGAAAGTAC
TAGCTTTTTTGCTAGCAGGCATGCTGGGGCCGCTCGAGTCTAGAGGGCCCGT
TTAAACCCGCTGATCAGCCTCGACTGTGCCTTCTAGTTGCCAGCCATCTGTTGT
TTGCCCCTCCCCCGTGCCTTCCTTGACCCTGGAAGGTGCCACTCCCACTGTCC
TTTCCTAATAAAATGAGGAAATTGCATCGCATTGTCTGAGTAGGTGTCATTCTAT
TCTGGGGGGTGGGGTGGGGCAGGACAGCAAGGGGGAGGATTGGGAAGACAA
TAGCAGGCATGCTGGGGATGCGGTGGGCTCTATGG

Second-generation iSBH-sgRNA 3 (CMV+MALAT1) trigger:
GACATTGATTATTGACTAGTTATTAATAGTAATCAATTACGGGGTCATTAGTTCAT
AGCCCATATATGGAGTTCCGCGTTACATAACTTACGGTAAATGGCCCGCCTGGC
TGACCGCCCAACGACCCCCGCCCATTGACGTCAATAATGACGTATGTTCCCATA
GTAACGCCAATAGGGACTTTCCATTGACGTCAATGGGTGGAGTATTTACGGTAA
ACTGCCCACTTGGCAGTACATCAAGTGTATCATATGCCAAGTACGCCCCCTATT
GACGTCAATGACGGTAAATGGCCCGCCTGGCATTATGCCCAGTACATGACCTT
ATGGGACTTTCCTACTTGGCAGTACATCTACGTATTAGTCATCGCTATTACCATG
GTGATGCGGTTTTGGCAGTACATCAATGGGCGTGGATAGCGGTTTGACTCACG
GGGATTTCCAAGTCTCCACCCCATTGACGTCAATGGGAGTTTGTTTTGGCACCA
AAATCAACGGGACTTTCCAAAATGTCGTAACAACTCCGCCCCATTGACGCAAAT
GGGCGGTAGGCGTGTACGGTGGGAGGTCTATATAAGCAGAGCTCTCTGGCTAA
CTAGAGAACCCACTGCTTACTGGCTTATCGAAATTAATACGACTCACTATAGGG
AGACCCAAGCTGGCTAGCGTTTAAACTTAAGGCTCGAAAGAGCCACTGTATTCA
CTGGTCCCATCCCACTCATTCCATACTTCCCGCCTCATGCTAGTACGAAAGTAC
TAGCTTTTTTGCTAGCAGGCATGCTGGGGCCGCGATTCGTCAGTAGGGTTGT
AAAGGTTTTTCTTTTCCTGAGAAAACAACCTTTTGTTTTCTCAGGTTTTGCTTTTT
GGCCTTTCCCTAGCTTTAAAAAAAAAAAAAGCAAAAGACGCTGGTGGCTGGCACT
CCTGGTTTCCAGGACGGGGTTCAAGTCCCTGCGGTGTCTTTGCTT

EYFP mRNA with 3' UTR trigger sequences

EYFP mRNA linker hairpin linker 100nt 5' flank linker 44nt trigger linker hairpin

EYFP mRNA + second-generation iSBH-sgRNA 1 trigger:
ATGAGCAGCGGCGCCCTGCTGTTCCACGGCAAGATCCCCTACGTGGTGGAGAT
GGAGGGCGATGTGGATGGCCACACCTTCAGCATCCGCGGTAAGGGCTACGGC
GATGCCAGCGTGGGCAAGGTGGATGCCCAGTTCATCTGCACCACCGGCGATG
TGCCCGTGCCCTGGAGCACCCTGGTGACCACCCTGACCTACGGCGCCCAGTG
CTTCGCCAAGTACGGCCCCGAGCTGAAGGATTTCTACAAGAGCTGCATGCCCG
ATGGCTACGTGCAGGAGCGCACCATCACCTTCGAGGGCGATGGCAATTTCAAG
ACCCGCGCCGAGGTGACCTTCGAGAATGGCAGCGTGTACAATCGCGTGAAGCT
GAATGGCCAGGGCTTCAAGAAGGATGGCCACGTGCTGGGCAAGAATCTGGAG
TTCAATTTCACCCCCCACTGCCTGTACATCTGGGGCGATCAGGCCAATCACGG
CCTGAAGAGCGCCTTCAAGATCTGCCACGAGATCGCCGGCAGCAAGGGCGATT
TCATCGTGGCCGATCACACCCAGATGAATACCCCCATCGGCGGCGGCCCCGT
GCACGTGCCCGAGTACCACCACATGAGCTACCACGTGAAGCTGAGCAAGGATG
TGACCGATCACCGCGATAATATGAGCCTGACGGAGACCGTGCGCGCCGTGGAT
TGCCGCAAGACCTACCTGTAACTCGAGTCTAGACCGGCTCGAAAGAGCCACCC
TATGGTGCACTCTCAGTACAATCTGCTCTGATGCCGCATAGTTAAGCCAGTATC
TGCTCCCTGCTTGTGTGTTGGAGGTCGCTGAGTAGTGCGCGAGCGTCAGTATG
GTCTTGGGATGCGTAGTGTGAACGTACGGACAGAAGCGACTAGCGAAGCAGC
GGCCGCATGCTAGTACGAAAGTACT

EYFP mRNA + second-generation iSBH-sgRNA 2 trigger:
ATGAGCAGCGGCGCCCTGCTGTTCCACGGCAAGATCCCCTACGTGGTGGAGAT
GGAGGGCGATGTGGATGGCCACACCTTCAGCATCCGCGGTAAGGGCTACGGC
GATGCCAGCGTGGGCAAGGTGGATGCCCAGTTCATCTGCACCACCGGCGATG
TGCCCGTGCCCTGGAGCACCCTGGTGACCACCCTGACCTACGGCGCCCAGTG
CTTCGCCAAGTACGGCCCCGAGCTGAAGGATTTCTACAAGAGCTGCATGCCCG
ATGGCTACGTGCAGGAGCGCACCATCACCTTCGAGGGCGATGGCAATTTCAAG
ACCCGCGCCGAGGTGACCTTCGAGAATGGCAGCGTGTACAATCGCGTGAAGCT
GAATGGCCAGGGCTTCAAGAAGGATGGCCACGTGCTGGGCAAGAATCTGGAG
TTCAATTTCACCCCCCACTGCCTGTACATCTGGGGCGATCAGGCCAATCACGG
CCTGAAGAGCGCCTTCAAGATCTGCCACGAGATCGCCGGCAGCAAGGGCGATT
TCATCGTGGCCGATCACACCCAGATGAATACCCCCATCGGCGGCGGCCCCGT
GCACGTGCCCGAGTACCACCACATGAGCTACCACGTGAAGCTGAGCAAGGATG
TGACCGATCACCGCGATAATATGAGCCTGACGGAGACCGTGCGCGCCGTGGAT
TGCCGCAAGACCTACCTGTAACTCGAGTCTAGACCGGCTCGAAAGAGCCACGC
AAAATTTAAGCTACAACAAGGCAAGGCTTGACCGACAATTGCATGAAGAATCTG
CTTAGGGTTAGGCGTTTTGCGCTGCTTCGCGATGTACGGGCCAGGTCATAGTA
TATGTGTGTTAGGGATGTTCGACTAAGCTGGTATCTCGCTGTACTGTCCTGCGG
CCGCATGCTAGTACGAAAGTACT

EYFP mRNA + second-generation iSBH-sgRNA 3 trigger:
ATGAGCAGCGGCGCCCTGCTGTTCCACGGCAAGATCCCCTACGTGGTGGAGAT
GGAGGGCGATGTGGATGGCCACACCTTCAGCATCCGCGGTAAGGGCTACGGC
GATGCCAGCGTGGGCAAGGTGGATGCCCAGTTCATCTGCACCACCGGCGATG
TGCCCGTGCCCTGGAGCACCCTGGTGACCACCCTGACCTACGGCGCCCAGTG
CTTCGCCAAGTACGGCCCCGAGCTGAAGGATTTCTACAAGAGCTGCATGCCCG
ATGGCTACGTGCAGGAGCGCACCATCACCTTCGAGGGCGATGGCAATTTCAAG
ACCCGCGCCGAGGTGACCTTCGAGAATGGCAGCGTGTACAATCGCGTGAAGCT
GAATGGCCAGGGCTTCAAGAAGGATGGCCACGTGCTGGGCAAGAATCTGGAG
TTCAATTTCACCCCCCACTGCCTGTACATCTGGGGCGATCAGGCCAATCACGG
CCTGAAGAGCGCCTTCAAGATCTGCCACGAGATCGCCGGCAGCAAGGGCGATT
TCATCGTGGCCGATCACACCCAGATGAATACCCCCATCGGCGGCGGCCCCGT
GCACGTGCCCGAGTACCACCACATGAGCTACCACGTGAAGCTGAGCAAGGATG
TGACCGATCACCGCGATAATATGAGCCTGACGGAGACCGTGCGCGCCGTGGAT
TGCCGCAAGACCTACCTGTAACTCGAGTCTAGACCGGCTCGAAAGAGCCACGT
CGACGGATCGGGAGATCTCCCGATCCCCTATGGTGCACTCTCAGTACAATCTG
CTCTGATGCCGCATAGTTAAGCCAGTATCTGCTCCCTGCTTGTGTGTCATCGGT
CTTGTCTGTATTCACTGGTCCCATCCCACTCATTCCATACTTCCCGCCTCGCGG
CCGCATGCTAGTACGAAAGTACT

Truncated RNA triggers for second-generation iSBH-sgRNAs

hairpin linker trigger randomized nt linker hairpin

trigger + randomized nt= 44nt

Second-generation iSBH-sgRNA 1 trigger (D1):
GGCTCGAAAGAGCCACGGATGCGTAGTGTGAACGTACGGAATCCGTGATACTA
ACGCCGGATGCTAGTACGAAAGTACTAGC

Second-generation iSBH-sgRNA 1 trigger (D2):
GGCTCGAAAGAGCCACGGATGCGTAGTGTGAACGTACGGACAGAATGATACTA

ACGCCGGATGCTAGTACGAAAGTACTAGC

Second-generation iSBH-sgRNA 2 trigger (D2):
GGCTCGAAAGAGCCACTGTTAGGGATGTTCGACTAAGCTGACGCGAATTGCGC
TGCAATAATGCTAGTACGAAAGTACTAGC

Second-generation iSBH-sgRNA 2 trigger (D1):
GGCTCGAAAGAGCCACTGTTAGGGATGTTCGACTAAGCTGGTATCAATTGCGC
TGCAATAATGCTAGTACGAAAGTACTAGC

Second-generation iSBH-sgRNA 3 trigger (D1):
GGCTCGAAAGAGCCACTGTATTCACTGGTCCCATCCCACTATGAATCGCTGAGA
TAGGAAATGCTAGTACGAAAGTACTAGC

Second-generation iSBH-sgRNA 4 trigger (D2):
GGCTCGAAAGAGCCACTGTATTCACTGGTCCCATCCCACTCATTCTCGCTGAGA
TAGGAAATGCTAGTACGAAAGTACTAGC

Endogenous trigger sequences for modular iSBH-sgRNA designs

hairpin linker endogenous trigger sequence linker hairpin

RNA trigger A:
GGCTCGAAAGAGCCACCTGATGATCAGTTTAGTCGTGTTGTGGTGATGATCAGT
TTAGTTGTGTTGTGGTGATGATCAGTTTAGTTGTGTTGTGGTGATGATCAGTTTA
GTCGTGTTGTGGTGATGATCAGTTTAGTCGTGTTGTGGTGATGATCTGTTTAGA
TGCTAGTACGAAAGTACTAGC

RNA trigger B:
GGCTCGAAAGAGCCACCTCAACAAAAGGTTAAAAATGGTTTAGTCACATGTATG
CTAACTTTCAATCTACAAAACTACAACCATCATCATAATCCCACAATCCTTCCTTC
CTCTGTTTCACTTCATCTGCCCCCATCAAAGAAAGCAGGGGATGACTAGAGACA
TTTGGTCGTTGTGATCGCCCGCCGTGAAATTGGCCCTTGGGCACGGATGATCG
GACGTAAGTCTCAAACGCCGGTTGGTGAGCTGTGAGGTGTTGGATTTCTGCGT
TTATTACTGATAATGCTAGTACGAAAGTACTAGC

RNA trigger C:
GGCTCGAAAGAGCCACTCTTAAAGTGCCAGCTTTCCCCTTGAAGTTCTGCCTAA
GGTCATCCCTCCTCCCTCGCCTCTCCTTTCACAGTCTGTCTGTCAAAGTCCCGT
CAGTCAGCCCGAGCCCGCGCCCCCGCCCCTGCACGCGCCTCGGGCACTCGC
GTTCTGTCGCTCTGGAATGAGCGAGCGAGCGAGAGAGAGAGGCGCGCTGCGC
ACTCCCGACTGGACAACAAGACTGTTTGTGGTTCTGAGACGCGAAAGCGAATG
TGGGTCAAAATCTGACAAGATGCTAGTACGAAAGTACTAGC

RNA trigger D:
GGCTCGAAAGAGCCACCACTGGCTTAGGAGTCTCAGCTCTGGGTACTCCCTCT
GAATAATGTTTGTCCTTATCTGTGCAGAGAACACTGTCTCTAAAGCATCCTTTGG
CAACACATTTGCTCAATCAACTACTGAATTGGTTGTTAAAATTAATTTTCCTTTAT
GCTAGTACGAAAGTACTAGC

Chemically modified sgRNAs and iSBH-sgRNAs tested in zebrafish

GC spacer* extension*loop extensionspacer scaffoldextra Us
Backfold=spacer*+extension+loop

*An additional GC sequence was added to all iSBH-sgRNA sequences to maintain consistency.

sgRNA 2 with chemical modifications
mG*mU*mA*rArGrUrCrGrGrArGrUrArCrUrGrUrCrCrUrGrUrUrUrArGrArGrCrUrAr
GrArArArUrArGrCrArArGrUrUrArArArArUrArArGrGrCrUrArGrUrCrCrGrUrUmAmUm
CmAmAmCmUmUmGmAmAmAmAmAmGmUmGmGmCmAmCmCmGmAmGmUm
CmGmGmUmGmCmU*mU*mU*rU

Second-generation iSBH-sgRNA 2 with chemical modifications (first iteration):
mG*mC*mA*rGrGrArCrArGrUrArCrArGrCrGrArGrArUrArCrCrArGrCrUrUrArGrUrCrG
rArArCrArUrCrCrCrUrArArCrArGrArCrArUrArGrCrCrCrGrUrArArGrUrCrGrGrArGrUr
ArCrUrGrUrCrCrUrGrUrUrUrArGrArGrCrUrArGrArArArUrArGrCrArArGrUrUrArArA
rArUrArArGrGrCrUrArGrUrCrCrGrUrUmAmUmCmAmAmCmUmUmGmAmAmAmA
mAmGmUmGmGmCmAmCmCmGmAmGmUmCmGmGmUmGmCmU*mU*mU*rU

Second-generation iSBH-sgRNA 2 with chemical modifications (second iteration):
mG*mC*mA*rGrGrArCrArGrUrArCrArGrCrGrArGrArUrArCrCrArGrCrUrUrArGrUrCrG
rArArCrArUrCrCrCrUrArArCrArGrArCrArUrArGrCrCrCmG*mU*mA*rArGrUrCrGrGrAr
GrUrArCrUrGrUrCrCrUrGrUrUrUrArGrArGrCrUrArGrArArArUrArGrCrArArGrUrUrA
rArArArUrArArGrGrCrUrArGrUrCrCrGrUrUmAmUmCmAmAmCmUmUmGmAmAmA
mAmAmGmUmGmGmCmAmCmCmGmAmGmUmCmGmGmUmGmCmU*mU*mU*rU

m= 2’OMe RNA base
r= ribobase
*= phosphorothioate bond

Chemically modified RNA triggers tested in zebrafish

hairpin linker 34nt trigger 100nt 3’ flank linker hairpin
random sequence

Non-complementary RNA trigger with chemical modifications:
mG*mG*mC*rUrCrGrArArArGrArGrCrCrArCrGrGrGrUrCrUrUrCrGrArGrCrArUrUrGr
GrArArGrUrCrUrArGrArUrArCrCrUrGrCrUrUrArCrGrArArGrArCrCrUrArUrGrCrUrA
rGrUrArCrGrArArArGrUrArCrU*mA*mG*mC

Second-generation iSBH-sgRNA 2 trigger with chemical modifications:
mG*mG*mC*rUrCrGrArArArGrArGrCrCrArCrUrGrUrUrArGrGrGrArUrGrUrUrCrGrAr
CrUrArArGrCrUrGrGrUrArUrCrUrCrGrCrUrGrUrArCrUrGrUrCrCrUrArUrGrCrUrArGr
UrArCrGrArArArGrUrArCrU*mA*mG*mC

m= 2’OMe RNA base
r= ribobase
*= phosphorothioate bond

1xCTS sequences

sequence matching sgRNA PAM site

1xCTS sgRNA 1: CAGTCGCGTGTAGCGAAGCAAGG

1xCTS sgRNA 2: GTAAGTCGGAGTACTGTCCTAGG

1xCTS sgRNA 3: CATGACATTATTCCCGCCTCAGG

1xCTS sgRNA 4: CCAGGTAATGACTAGCAGGAAGG

1xCTS sgRNA 5: GCACTACTTCAGTAAGAGTCAGG

8xCTS sequences

Linker 8x(sequence matching sgRNA PAM site linker) linker

8xCTS sgRNA 1:
TCTAGAGAGCTCCAGTCGCGTGTAGCGAAGCACGGCTATGAAAATATTAACAGT
CGCGTGTAGCGAAGCAGGGACTAGTAGGGTCAGTAGGAGCTCCAGTCGCGTG
TAGCGAAGCACGGCTATGAAAATATTAACAGTCGCGTGTAGCGAAGCAGGGAC
TAGTGGGCCCCTAGAGAGCTCCAGTCGCGTGTAGCGAAGCACGGCTATGAAAA
TATTAACAGTCGCGTGTAGCGAAGCAGGGACTAGTAGGGTCAGTAGGAGCTCC
AGTCGCGTGTAGCGAAGCACGGCTATGAAAATATTAACAGTCGCGTGTAGCGA
AGCAGGGACTAGTGCTAGC

8xCTS sgRNA 2:
TCTAGAGAGCTCGTAAGTCGGAGTACTGTCCTCGGCTATGAAAATATTAAGTAA
GTCGGAGTACTGTCCTGGGACTAGTAGGGTCAGTAGGAGCTCGTAAGTCGGAG
TACTGTCCTCGGCTATGAAAATATTAAGTAAGTCGGAGTACTGTCCTGGGACTA
GTGGGCCCCTAGAGAGCTCGTAAGTCGGAGTACTGTCCTCGGCTATGAAAATA
TTAAGTAAGTCGGAGTACTGTCCTGGGACTAGTAGGGTCAGTAGGAGCTCGTA
AGTCGGAGTACTGTCCTCGGCTATGAAAATATTAAGTAAGTCGGAGTACTGTCC
TGGGACTAGTGCTAGC

8xCTS sgRNA 3:
TCTAGAGAGCTCCATGACATTATTCCCGCCTCCGGCTATGAAAATATTAACATG
ACATTATTCCCGCCTCGGGACTAGTAGGGTCAGTAGGAGCTCCATGACATTATT
CCCGCCTCCGGCTATGAAAATATTAACATGACATTATTCCCGCCTCGGGACTAG
TGGGCCCCTAGAGAGCTCCATGACATTATTCCCGCCTCCGGCTATGAAAATATT
AACATGACATTATTCCCGCCTCGGGACTAGTAGGGTCAGTAGGAGCTCCATGAC
ATTATTCCCGCCTCCGGCTATGAAAATATTAACATGACATTATTCCCGCCTCG
GGACTAGTGCTAG

8xCTS sgRNA 4:
TCTAGAGAGCTCCCAGGTAATGACTAGCAGGACGGCTATGAAAATATTAACCAG
GTAATGACTAGCAGGAGGGACTAGTAGGGTCAGTAGGAGCTCCCAGGTAATGA
CTAGCAGGACGGCTATGAAAATATTAACCAGGTAATGACTAGCAGGAGGGACT
AGTGGGCCCCTAGAGAGCTCCCAGGTAATGACTAGCAGGACGGCTATGAAAAT
ATTAACCAGGTAATGACTAGCAGGAGGGACTAGTAGGGTCAGTAGGAGCTCCC
AGGTAATGACTAGCAGGACGGCTATGAAAATATTAACCAGGTAATGACTAGCAG
GAGGGACTAGTGCTAG

8xCTS sgRNA 5:
TCTAGAGAGCTCGCACTACTTCAGTAAGAGTCCGGCTATGAAAATATTAAGCAC
TACTTCAGTAAGAGTCGGGACTAGTAGGGTCAGTAGGAGCTCGCACTACTTCA

GTAAGAGTCCGGCTATGAAAATATTAAGCACTACTTCAGTAAGAGTCGGGACTA
GTGGGCCCCTAGAGAGCTCGCACTACTTCAGTAAGAGTCCGGCTATGAAAATA
TTAAGCACTACTTCAGTAAGAGTCGGGACTAGTAGGGTCAGTAGGAGCTCGCA
CTACTTCAGTAAGAGTCCGGCTATGAAAATATTAAGCACTACTTCAGTAAGAGTC
GGGACTAGTGCTAG

*8xCTS sequences displayed here are in reverse orientation

Oligos and primer sequences ordered for cloning 8xCTS reporters

Oligo 2xCTS sgRNA 1:
GGACCCTACTAGTCCCTGCTTCGCTACACGCGACTGTTAATATTTTCATAGCCG
TGCTTCGCTACACGCGACTGGAGCTCCTACTGACCC

Oligo 2xCTS sgRNA 2:
GGACCCTACTAGTCCCAGGACAGTACTCCGACTTACTTAATATTTTCATAGCCG
AGGACAGTACTCCGACTTACGAGCTCCTACTGACCC

Oligo 2xCTS sgRNA 3:
GGACCCTACTAGTCCCGAGGCGGGAATAATGTCATGTTAATATTTTCATAGCCG
GAGGCGGGAATAATGTCATGGAGCTCCTACTGACCC

Oligo 2xCTS sgRNA 4:
GGACCCTACTAGTCCCTCCTGCTAGTCATTACCTGGTTAATATTTTCATAGCCGT
CCTGCTAGTCATTACCTGGGAGCTCCTACTGACCC

Oligo 2xCTS sgRNA 5:
GGACCCTACTAGTCCCGACTCTTACTGAAGTAGTGCTTAATATTTTCATAGCCG
GACTCTTACTGAAGTAGTGCGAGCTCCTACTGACCC

<u>Primers used for amplifying 2xCTS oligos and cloning 4xCTS-ECFP reporters</u>
OP_2xCTS_FW: ACTGCCGTCTCGGACCCTACTAGTCCC
OP_2xCTS_RW: ACCTACGTCTCGGGTCAGTAGGAGCTC

<u>Primers used for amplifying 4xCTS plasmids and cloning 8xCTS-ECFP reporters</u>
OP_4xCTS_NheI_FW: AGAGATACCGGGCGCTAGCACTAGTCCC
OP_4xCTS_ApaI_RW: GCCCCCCGCTAGTGGGCCCCTAGAGAGCTC

Oligos and primer sequences ordered for repetitive trigger sequences

<u>Trigger A</u>

OP_trig_A_oligo_1:
GAAGAGGAAGACCTCCACCTGATGATCAGTTTAGTCGTGTTGTGGTGATGATC
AGTTTAGTTGTGTTGCTGTCTTCGCACTACGC

OP_trig_A_oligo_1_FW_BbsI: GAAGAGGAAGACCTCCAC
OP_trig_A_oligo_1+2_RW_BbsI: GCGTAGTGCGAAGACAG

OP_trig_A_oligo_2:
CCACGACTGGAAGACTGGTTGTGGTGATGATCAGTTTAGTTGTGTTGTGGTGA
TGATCAGTTTAGTCCTGTCTTCGCACTACGC

OP_trig_A_oligo_2+3_FW_BbsI: CCACGACTGGAAGACTG

OP_trig_A_oligo_1+2_RW_BbsI: GCGTAGTGCGAAGACAG

OP_trig_A_oligo_3:
CCACGACTGGAAGACTGAGTCGTGTTGTGGTGATGATCAGTTTAGTCGTGTTG TGGTGATGATCTGTTTAGATGCCGGTCTTCTCGTC

OP_trig_A_oligo_2+3_FW_BbsI: CCACGACTGGAAGACTG
OP_trig_A_oligo_3_RW_BbsI: GACGAGAAGACCGGCAT

Trigger B

OP_trig_B_oligo_1:
CATGTATGCTAACTTTCAATCTACAAAACTACAACCATCATCATAATCCCACAA TCCTTCCTTCCTCTGTTTTTCACTTCATCTGCCCCC

OP_trig_B_oligo_1_FW_BbsI:
AAGAGGAAGACCTCCACCTCAACAAAAGGTTAAAAATGGTTTAGTCACATGTAT GCTAACTTTCAATCTAC

OP_trig_B_oligo_1_RW_BbsI:
ATGCAGAAGACCACCTGCTTTCTTTGATGGGGGCAGATGAAGTGAAAAAC

OP_trig_B_oligo_2:
CATTTGGTCGTTGTGATCGCCCGCCGTGAAATTGGCCCTTGGGCACGGATGAT CGGACGTAAGTCTCAAACGCCGGTTGGTGAGCTGTGA

OP_trig_B_oligo_2_FW_BbsI:
ACTGGAAGACTGCAGGGGATGACTAGAGACATTTGGTCGTTGTGATCG

OP_trig_B_oligo_2_RW_BbsI:
TACTAGAAGACTGGCATTATCAGTAATAAACGCAGAAATCCAACACCTCACAGC TCACCAACC

Primer sequences for RNA circularisation assays

Second-generation iSBH-sgRNA circularisation assay

Second-generation iSBH-sgRNA 1 primers

OP_Ca_nes1_F: TTATCAACTTGAAAAAGTGGCAC
OP_Ca_nes1_R+RT: GACTAGCCTTATTTTAACTTGC
(primer also used for RT)
OP_CA_nes2_NheI_F: AAGCTGGCTAGCAAAAGTGGCACCGAGTC
OP_CA_nes2_sgRNA1_NotI_R: ACTCGAGCGGCCGCCTAGCTCTAAAACTGCTTCG

Second-generation iSBH-sgRNA 2 primers

OP_Ca_nes1_F: TTATCAACTTGAAAAAGTGGCAC
OP_Ca_nes1_R+RT: GACTAGCCTTATTTTAACTTGC
(primer also used for RT)
OP_CA_nes2_NheI_F: AAGCTGGCTAGCAAAAGTGGCACCGAGTC

OP_CA_ nes2_sgRNA2_NotI_R: ACTCGAGCGGCCGCCTAGCTCTAAAACAGGACAG

Second-generation iSBH-sgRNA 3 primers

OP_Ca_nes1_F: TTATCAACTTGAAAAAGTGGCAC
OP_Ca_nes1_R+RT: GACTAGCCTTATTTTAACTTGC
(primer also used for RT)
OP_CA_nes2_NheI_F: AAGCTGGCTAGCAAAAGTGGCACCGAGTC
OP_CA_nes2_sgRNA3_NotI_R: ACTCGAGCGGCCGCCTAGCTCTAAAACGAGGCG

RNA triggers circularisation assay

Second-generation iSBH-sgRNA 1 trigger 100nt 3' flank primers

OP_CA_nes1_trig1_F: CCACCAAGGCAAAGAGAAG
OP_CA_nes1_trig1_R+RT: CTACTCCTAATGGTTCAATTTTTACTAC
(primer also used for RT)
OP_CA_nes2_trig1_F_NheI: AAGCTGGCTAGCCAAAGAGAAGAGTGGTGCAG
OP_CA_nes2_trig1_R_NotI:
ACTCGAGCGGCCGCCAATTTTTACTACTTTATATTTATATAATTCACTTCTC

Second-generation iSBH-sgRNA 2 trigger 100nt 3' flank primers

OP_CA_nes1_trig2_F: TACAACAAGGCAAGGCTTG
OP_CA_nes1_trig2_R+RT: CTTAAATTTTGCTCGCGCAC
(primer also used for RT)
OP_CA_nes2_trig2_F_NheI: AAGCTGGCTAGCCAAGGCTTGACCGACAAG
OP_CA_nes2_trig2_R_BbsI: GAGCCGAAGACCCGGCCGCCGACCTCCAACACACAAG

Second-generation iSBH-sgRNA 3 trigger 100nt 3' flank primers

OP_CA_nes1_trig3_F: AGTATCTGCTCCCTGCTTG
OP_CA_nes1_trig3_R+RT: TTAACTATGCGGCATCAGAG
(primer also used for RT)
OP_CA_nes2_trig3_F_NheI: AAGCTGGCTAGCGCTTGTGTGTTGGAGGTG
OP_CA_nes2_trig3_R_NotI: ACTCGAGCGGCCGCGAGCAGATTGTACTGAGAGTG

Primer sequences- zebrafish genotyping

OP_dCas9_Vp64_nes1_F: AAATAAGCGAATTCTCCAAAAG
OP_dCas9_Vp64_nes1_R:CTACGTCTCGCCGCATGTTAGCAGACTTCCTCTGCC
CTCCGAGCCACCGCCCTTGTACAGCTCGTCCATG
OP_dCas9_Vp64_nes2_F: TGCTTGGTTCGGATGCCCTTGATGAC
OP_dCas9_Vp64_nes2_R: GGCGAAGCACATCAGGCCGTAGC
OP_8xCTS _nes1_F: GGTACGGGAGGTACTTGGAGCG
OP_8xCTS _nes1_R: CACCACCCCGGTGAACAGCTC
OP_8xCTS _nes2_F: CTTGGAGCGGCCGCAATAAATATC
OP_8xCTS _nes2_R: CTTGCTCACCATGGTCAACGC
